# Supplementary material for: Chromothripsis during telomere crisis is independent of NHEJ, and consistent with a replicative origin
Source: Genome Res. 2019 May;29(5):737–49. doi: 10.1101/gr.240705.118 (PMC6499312; doi:10.1101/gr.240705.118)
Supplement: Supplemental Material [file supp_gr.240705.118_Supplemental_file_1.zip › contigs/annotated_contigs/DB57/contig.2.DB57_length_488_mean_cov_2.76639344262.docx]

**DB57_length_488_mean_cov_2.76639344262**

```
TCATCTTAGAAGTTTTAGGTGTGATCTGGCTTCCTGGTAACATCAGAATGCTCTAGGAGTTGCCCAGTTTCCAGATACTAGACTTATTG
 >chr1:174552806-174552910 + E=5e-51
CCCTTGTGCT|GTAGA|CCATATGATCATTTAAATAGAAACAAAAAAACCATTCAATAAAATTCACCATCTCTTCATGATTAAATTTAA
           >chr1:174565735-174566124 - E=1e-210
AAAAACCTAAAAAAACTGGATAGAGAAGAAACATACTTCAACACACAATAAAAGCCATATAGAAAAGAACCACATCTACATATGCGTAA

AACCTGAAAATCTTTCCTCCAAGATCTGGAACAAGACAAAGATGCCCACTTTCACCACCGTTATTCAACACAGTCCTGGAAATCCTAGC

TAAAGGAATCAGATAAGATAAAGAAATAAAGGTCATCCAAACTGAAATGGAAGAAGTCAAATTATCCTTCTTTGCAGATGATATAATCT

TATATTTAGAAAAACCTAAAGACTTAATCAAGAAATTATTAGAAC
```
